# Does a perceptual gap lead to actions against digital misinformation? A third-person effect study among medical students

**DOI:** 10.1186/s12889-024-18763-9

**Published:** 2024-05-11

**Authors:** Zongya Li, Jun Yan

**Affiliations:** Journalism and Information Communication School, Huazhong University of Science and Technology, Wuhan, Hubei China

**Keywords:** Digital misinformation, Third-person perception, Pre-professionals, Efficacy, Collectivism, Professional identification

## Abstract

**Background:**

We are making progress in the fight against health-related misinformation, but mass participation and active engagement are far from adequate. Focusing on pre-professional medical students with above-average medical knowledge, our study examined whether and how third-person perceptions (TPP), which hypothesize that people tend to perceive media messages as having a greater effect on others than on themselves, would motivate their actions against misinformation.

**Methods:**

We collected the cross-sectional data through a self-administered paper-and-pencil survey of 1,500 medical students in China during April 2022.

**Results:**

Structural equation modeling (SEM) analysis, showed that TPP was negatively associated with medical students’ actions against digital misinformation, including rebuttal of misinformation and promotion of corrective information. However, self-efficacy and collectivism served as positive predictors of both actions. Additionally, we found professional identification failed to play a significant role in influencing TPP, while digital misinformation self-efficacy was found to broaden the third-person perceptual gap and collectivism tended to reduce the perceptual bias significantly.

**Conclusions:**

Our study contributes both to theory and practice. It extends the third-person effect theory by moving beyond the examination of restrictive actions and toward the exploration of corrective and promotional actions in the context of misinformation., It also lends a new perspective to the current efforts to counter digital misinformation; involving pre-professionals (in this case, medical students) in the fight.

## Introduction

The widespread persistence of misinformation in the social media environment calls for effective strategies to mitigate the threat to our society [1]. Misinformation has received substantial scholarly attention in recent years [2], and solution-oriented explorations have long been a focus but the subject remains underexplored [3].

Health professionals, particularly physicians and nurses, are highly expected to play a role in the fight against misinformation as they serve as the most trusted information sources regarding medical topics [4]. However, some barriers, such as limitations regarding time and digital skills, greatly hinder their efforts to tackle misinformation on social media [5].

Medical students (i.e., college students majoring in health/medical science), in contrast to medical faculty, have a greater potential to become the major force in dealing with digital misinformation as they are not only equipped with basic medical knowledge but generally possess greater social media skills than the former generation [6]. Few studies, to our knowledge, have tried to explore the potential of these pre-professionals in tackling misinformation. Our research thus fills the gap by specifically exploring how these pre-professionals can be motivated to fight against digital health-related misinformation.

The third-person perception (TPP), which states that people tend to perceive media messages as having a greater effect on others than on themselves [7], has been found to play an important role in influencing individuals’ coping strategies related to misinformation. But empirical exploration from this line of studies has yielded contradictory results. Some studies revealed that individuals who perceived a greater negative influence of misinformation on others than on themselves were more likely to take corrective actions to debunk misinformation [8]. In contrast, some research found that stronger TPP reduced individuals’ willingness to engage in misinformation correction [9, 10]. Such conflicting findings impel us to examine the association between the third-person perception and medical students’ corrective actions in response to misinformation, thus attempting to unveil the underlying mechanisms that promote or inhibit these pre-professionals’ engagement with misinformation.

Researchers have also identified several perceptual factors that motivate individuals’ actions against misinformation, especially efficacy-related concepts (e.g., self-efficacy and health literacy) and normative variables (e.g., subjective norms and perceived responsibility) [3, 8, 9]. However, most studies devote attention to the general population; little is known about whether and how these factors affect medical students’ intentions to deal with misinformation. We recruited Chinese medical students in order to study a social group that is mutually influenced by cultural norms (collectivism in Chinese society) and professional norms. Meanwhile, systematic education and training equip medical students with abundant clinical knowledge and good levels of eHealth literacy [5], which enable them to have potential efficacy in tackling misinformation. Our study thus aims to examine how medical students’ self-efficacy, cultural norms (i.e., collectivism) and professional norms (i.e., professional identification) impact their actions against misinformation.

Previous research has found self-efficacy to be a reliable moderator of optimistic bias, the tendency for individuals to consider themselves as less likely to experience negative events but more likely to experience positive events as compared to others [11–13]. As TPP is thought to be a product of optimistic bias, accordingly, self-efficacy should have the potential to influence the magnitude of third-person perception [14, 15]. Meanwhile, scholars also suggest that the magnitude of TPP is influenced by social distance corollary [16, 17]. Simply put, individuals tend to perceive those who are more socially distant from them to be more susceptible to the influence of undesirable media than those who are socially proximal [18–20]. From a social identity perspective, collectivism and professional identification might moderate the relative distance between oneself and others while the directions of such effects differ [21, 22]. For example, collectivists tend to perceive a smaller social distance between self and others as “they are less likely to view themselves as distinct or unique from others” [23]. In contrast, individuals who are highly identified with their professional community (i.e., medical community) are more likely to perceive a larger social distance between in-group members (including themselves) and out-group members [24]. In this way, collectivism and professional identification might exert different effects on TPP. On this basis, this study aims to examine whether and how medical students’ perceptions of professional identity, self-efficacy and collectivism influence the magnitude of TPP and in turn influence their actions against misinformation.

Our study builds a model that reflects the theoretical linkages among self-efficacy, collectivism, professional identity, TPP, and actions against misinformation. The model, which clarifies the key antecedents of TPP and examines the mediating role of TPP, contribute to the third-person effect literature and offer practical contributions to countering digital misinformation.

### Context of the study

As pre-professionals equipped with specialized knowledge and skills, medical students have been involved in efforts in health communication and promotion during the pandemic. For instance, thousands of medical students have participated in various volunteering activities in the fight against COVID-19, such as case data visualization [25], psychological counseling [26], and providing online consultations [27]. Due to the shortage of medical personnel and the burden of work, some medical schools also encouraged their students to participate in health care assistance in hospitals during the pandemic [28, 29].

The flood of COVID-19 related misinformation has posed an additional threat to and burden on public health. We have an opportunity to address this issue and respond to the general public’s call for guidance from the medical community about COVID-19 by engaging medical students as a main force in the fight against coronavirus related misinformation.

## Literature review

### The third-person effect in the misinformation context

Originally proposed by Davison [7], the third-person effect hypothesizes that people tend to perceive a greater effect of mass media on others than on themselves. Specifically, the TPE consists of two key components: the perceptual and the behavioral [16]. The perceptual component centers on the perceptual gap where individuals tend to perceive that others are more influenced by media messages than themselves. The behavioral component refers to the behavioral outcomes of the self-other perceptual gap in which people act in accordance with such perceptual asymmetry.

According to Perloff [30], the TPE is contingent upon situations. For instance, one general finding suggests that when media messages are considered socially undesirable, nonbeneficial, or involving risks, the TPE will get amplified [16]. Misinformation characterized as inaccurate, misleading, and even false, is regarded as undesirable in nature [31]. Based on this line of reasoning, we anticipate that people will tend to perceive that others would be more influenced by misinformation than themselves.

Recent studies also provide empirical evidence of the TPE in the context of misinformation [32]. For instance, an online survey of 511 Chinese respondents conducted by Liu and Huang [33] revealed that individuals would perceive others to be more vulnerable to the negative influence of COVID-19 digital disinformation. An examination of the TPE within a pre-professional group – the medical students–will allow our study to examine the TPE scholarship in a particular population in the context of tackling misinformation.

### Why TPE occurs among medical students: a social identity perspective

Of the works that have provided explanations for the TPE, the well-known ones include self-enhancement [34], attributional bias [35], self-categorization theory [36], and the exposure hypothesis [19]. In this study, we argue for a social identity perspective as being an important explanation for third-person effects of misinformation among medical students [36, 37].

The social identity explanation suggests that people define themselves in terms of their group memberships and seek to maintain a positive self-image through favoring the members of their own groups over members of an outgroup, which is also known as downward comparison [38, 39]. In intergroup settings, the tendency to evaluate their ingroups more positively than the outgroups will lead to an ingroup bias [40]. Such an ingroup bias is typically described as a trigger for the third-person effect as individuals consider themselves and their group members superior and less vulnerable to undesirable media messages than are others and outgroup members [20].

In the context of our study, medical students highly identified with the medical community tend to maintain a positive social identity through an intergroup comparison that favors the ingroup and derogates the outgroup (i.e., the general public). It is likely that medical students consider themselves belonging to the medical community and thus are more knowledgeable and smarter than the general public in health-related topics, leading them to perceive the general public as more vulnerable to health-related misinformation than themselves. Accordingly, we propose the following hypothesis:

*H1: As medical students’ identification with the medical community increases, the TPP concerning digital misinformation will become larger.*

### What influences the magnitude of TPP

Previous studies have demonstrated that the magnitude of the third-person perception is influenced by a host of factors including efficacy beliefs [3] and cultural differences in self-construal [22, 23]. Self-construal is defined as “a constellation of thoughts, feelings, and actions concerning the relationship of the self to others, and the self as distinct from others” [41]. Markus and Kitayama (1991) identified two dimensions of self-construal: Independent and interdependent. Generally, collectivists hold an interdependent view of the self that emphasizes harmony, relatedness, and places importance on belonging, whereas individualists tend to have an independent view of the self and thus view themselves as distinct and unique from others [42]. Accordingly, cultural values such as collectivism-individualism should also play a role in shaping third-person perception due to the adjustment that people make of the self-other social identity distance [22].

Set in a Chinese context aiming to explore the potential of individual-level approaches to deal with misinformation, this study examines whether collectivism (the prevailing cultural value in China) and self-efficacy (an important determinant of ones’ behavioral intentions) would affect the magnitude of TPP concerning misinformation and how such impact in turn would influence their actions against misinformation.

#### The impact of self-efficacy on TPP

Bandura [43] refers to self-efficacy as one’s perceived capability to perform a desired action required to overcome barriers or manage challenging situations. He also suggests understanding self-efficacy as “a differentiated set of self-beliefs linked to distinct realms of functioning” [44]. That is to say, self-efficacy should be specifically conceptualized and operationalized in accordance with specific contexts, activities, and tasks [45]. In the context of digital misinformation, this study defines self-efficacy as one’s belief in his/her abilities to identify and verify misinformation within an affordance-bounded social media environment [3].

Previous studies have found self-efficacy to be a reliable moderator of biased optimism, which indicates that the more efficacious individuals consider themselves, the greater biased optimism will be invoked [12, 23, 46]. Even if self-efficacy deals only with one’s assessment of self in performing a task, it can still create the other-self perceptual gap; individuals who perceive a higher self-efficacy tend to believe that they are more capable of controlling a stressful or challenging situation [12, 14]. As such, they are likely to consider themselves less vulnerable to negative events than are others [23]. That is, individuals with higher levels of self-efficacy tend to underestimate the impact of harmful messages on themselves, thereby widening the other-self perceptual gap.

In the context of fake news, which is closely related to misinformation, scholars have confirmed that fake news efficacy (i.e., a belief in one’s capability to evaluate fake news [3]) may lead to a larger third-person perception. Based upon previous research evidence, we thus propose the following hypothesis:

*H2: As medical students’ digital misinformation self-efficacy increases, the TPP concerning digital misinformation will become larger.*

#### The influence of collectivism on TPP

Originally conceptualized as a societal-level construct [47], collectivism reflects a culture that highlights the importance of collective goals over individual goals, defines the self in relation to the group, and places great emphasis on conformity, harmony and interdependence [48]. Some scholars propose to also examine cultural values at the individual level as culture is embedded within every individual and could vary significantly among individuals, further exerting effects on their perceptions, attitudes, and behaviors [49]. Corresponding to the construct at the macro-cultural level, micro-psychometric collectivism which reflects personality tendencies is characterized by an interdependent view of the self, a strong sense of other-orientation, and a great concern for the public good [50].

A few prior studies have indicated that collectivism might influence the magnitude of TPP. For instance, Lee and Tamborini [23] found that collectivism had a significant negative effect on the magnitude of TPP concerning Internet pornography. Such an impact can be understood in terms of biased optimism and social distance. Collectivists tend to view themselves as an integral part of a greater social whole and consider themselves less differentiated from others [51]. Collectivism thus would mitigate the third-person perception due to a smaller perceived social distance between individuals and other social members and a lower level of comparative optimism [22, 23]. Based on this line of reasoning, we thus propose the following hypothesis:

*H3: As medical students’ collectivism increases, the TPP concerning digital misinformation will become smaller.*

### Behavioral consequences of TPE in the misinformation context

The behavioral consequences trigged by TPE have been classified into three categories: restrictive actions refer to support for censorship or regulation of socially undesirable content such as pornography or violence on television [52]; corrective action is a specific type of behavior where people seek to voice their own opinions and correct the perceived harmful or ambiguous messages [53]; promotional actions target at media content with desirable influence, such as advocating for public service announcements [24]. In a word, restriction, correction and promotion are potential behavioral outcomes of TPE concerning messages with varying valence of social desirability [16].

Restrictive action as an outcome of third-person perceptual bias (i.e., the perceptual component of TPE positing that people tend to perceive media messages to have a greater impact on others than on themselves) has received substantial scholarly attention in past decades; scholars thus suggest that TPE scholarship to go beyond this tradition and move toward the exploration of corrective and promotional behaviors [16, 24]. Moreover, individual-level corrective and promotional actions deserve more investigation specifically in the context of countering misinformation, as efforts from networked citizens have been documented as an important supplement beyond institutional regulations (e.g., drafting policy initiatives to counter misinformation) and platform-based measures (e.g., improving platform algorithms for detecting misinformation) [8].

In this study, corrective action specifically refers to individuals’ reactive behaviors that seek to rectify misinformation; these include such actions as debunking online misinformation by commenting, flagging, or reporting it [3, 54]. Promotional action involves advancing correct information online, including in response to misinformation that has already been disseminated to the public [55].

#### The impact of TPP on corrective and promotional actions

Either paternalism theory [56] or the protective motivation theory [57] can act as an explanatory framework for behavioral outcomes triggered by third-person perception. According to these theories, people act upon TPP as they think themselves to know better and feel obligated to protect those who are more vulnerable to negative media influence [58]. That is, corrective and promotional actions as behavioral consequences of TPP might be driven by a protective concern for others and a positive sense of themselves.

To date, several empirical studies across contexts have examined the link between TPP and corrective actions. Koo et al. [8], for instance, found TPP was not only positively related to respondents’ willingness to correct misinformation propagated by others, but also was positively associated with their self-correction. Other studies suggest that TPP motivates individuals to engage in both online and offline corrective political participation [59], give a thumbs down to a biased story [60], and implement corrective behaviors concerning “problematic” TV reality shows [16]. Based on previous research evidence, we thus propose the following hypothesis:

*H4: Medical students with higher degrees of TPP will report greater intentions to correct digital misinformation.*

Compared to correction, promotional behavior has received less attention in the TPE research. Promotion commonly occurs in a situation where harmful messages have already been disseminated to the public and others appear to have been influenced by these messages, and it serves as a remedial action to amplify messages with positive influence which may in turn mitigate the detrimental effects of harmful messages [16].

Within this line of studies, however, empirical studies provide mixed findings. Wei and Golan [24] found a positive association between TPP of desirable political ads and promotional social media activism such as posting or linking the ad on their social media accounts. Sun et al. [16] found a negative association between TPP regarding clarity and community-connection public service announcements (PSAs) and promotion behaviors such as advocating for airing more PSAs in TV shows.

As promotional action is still underexplored in the TPE research, and existing evidence for the link between TPP and promotion is indeed mixed, we thus propose an exploratory research question:

*RQ1: What is the relationship between TPP and medical students’ intentions to promote corrective information?*

### The impact of self-efficacy and collectivism on actions against misinformation

According to social cognitive theory, people with higher levels of self-efficacy tend to believe they are competent and capable and are more likely to execute specific actions [43]. Within the context of digital misinformation, individuals might become more willing to engage in misinformation correction if they have enough knowledge and confidence to evaluate information, and possess sufficient skills to verify information through digital tools and services [61].

Accordingly, we assumed medical students with higher levels of digital misinformation self-efficacy would be likely to become more active in the fight against misinformation.

*H5: Medical students with higher levels of digital misinformation self-efficacy will report greater intentions to (a) correct misinformation and (b) promote corrective information on social media.*

Social actions of collectivists are strongly guided by prevailing social norms, collective responsibilities, and common interest, goals, and obligations [48]. Hence, highly collectivistic individuals are more likely to self-sacrifice for group interests and are more oriented toward pro-social behaviors, such as adopting pro-environmental behaviors [62], sharing knowledge [23], and providing help for people in need [63].

Fighting against misinformation is also considered to comprise altruism, especially self-engaged corrective and promotional actions, as such actions are costly to the actor (i.e., taking up time and energy) but could benefit the general public [61]. Accordingly, we assume collectivism might play a role in prompting people to engage in reactive behaviors against misinformation.

It is also noted that collectivist values are deeply rooted in Chinese society and were especially strongly advocated during the outbreak of COVID-19 with an attempt to motivate prosocial behaviors [63]. Accordingly, we expected that the more the medical students were oriented toward collectivist values, the more likely they would feel personally obliged and normatively motivated to engage in misinformation correction. However, as empirical evidence was quite limited, we proposed exploratory research questions:

*RQ2: Will medical students with higher levels of collectivism report greater intentions to (a) correct misinformation and (b) promote corrective information on social media?*

### The theoretical model

**Fig. 1:**
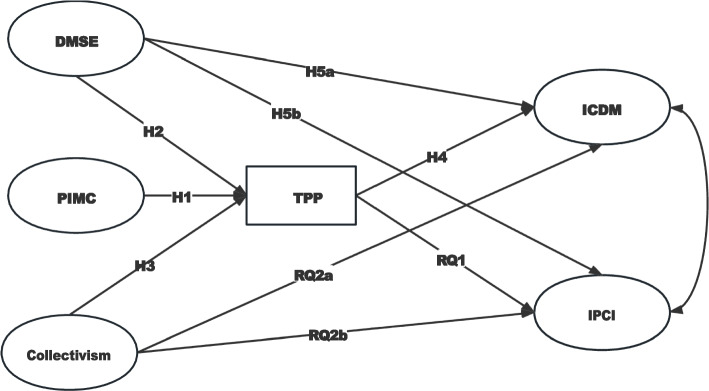
The proposed theoretical model. DMSE = Digital Misinformation Self-efficacy; PIMC = Professional Identification with Medical Community; ICDM = Intention to Correct Digital Misinformation; IPCI = Intention to Promote Corrective Information

To integrate both the antecedents and consequences of TPP, we proposed a theoretical model (as shown in Fig. 1) to examine how professional identification, self-efficacy and collectivism would influence the magnitude of TPP, and how such impact would in turn influence medical students’ intentions to correct digital misinformation and promote corrective information. Thus, RQ3 was proposed:

*RQ3: Will the TPP mediate the impact of self-efficacy and collectivism on medical students’ intentions to (a) correct misinformation, and (b) promote corrective information on social media?*

## Method

### Sampling

To examine the proposed hypotheses, this study utilized cross-sectional survey data from medical students in Tongji Medical College (TJMC) of China. TJMC is one of the birthplaces of Chinese modern medical education and among the first universities and colleges that offer eight-year curricula on clinical medicine. Further, TJMC is located in Wuhan, the epicenter of the initial COVID-19 outbreaks, thus its students might find the pandemic especially relevant – and threatening – to them.

The survey instrument was pilot tested using a convenience sample of 58 respondents, leading to minor refinements to a few items. Upon approval from the university’s Institutional Research Board (IRB), the formal investigation was launched in TJMC during April 2022. Given the challenges of reaching the whole target population and acquiring an appropriate sampling frame, this study employed purposive and convenience sampling.

We first contacted four school counselors as survey administrators through email with a letter explaining the objective of the study and requesting cooperation. All survey administrators were trained by the principal investigator to help with the data collection in four majors (i.e., basic medicine, clinical medicine, nursing, and public health). Paper-and-pencil questionnaires were distributed to students on regular weekly departmental meetings of each major as students in all grades (including undergraduates, master students, and doctoral students) were required to attend the meeting. The projected time of completion of the survey was approximately 10–15 min. The survey administrators indicated to students that participation was voluntary, their responses would remain confidential and secure, and the data would be used only for academic purposes. Though a total of 1,500 participants took the survey, 17 responses were excluded from the analysis as they failed the attention filters. Ultimately, a total of 1,483 surveys were deemed valid for analysis.

Of the 1,483 respondents, 624 (42.10%) were men and 855 (57.70%) were women, and four did not identify gender. The average age of the sample was 22.00 (*SD* = 2.54, ranging from 17 to 40). Regarding the distribution of respondents’ majors, 387 (26.10%) were in basic medicine, 390 (26.30%) in clinical medicine, 307 (20.70%) in nursing, and 399 (26.90%) in public health. In terms of university class, 1,041 (70.40%) were undergraduates, 291 (19.70%) were working on their master degrees, 146 (9.90%) were doctoral students, and five did not identify their class data.

### Measurement of key variables

#### Perceived effects of digital misinformation on oneself and on others

Three modified items adapted from previous research [33, 64] were employed to measure perceived effects of digital misinformation on oneself. Respondents were asked to indicate to what extent they agreed with the following: (1) I am frequently concerned that the information about COVID-19 I read on social media might be false; (2) Misinformation on social media might misguide my understanding of the coronavirus; (3) Misinformation on social media might influence my decisions regarding COVID-19. The response categories used a 7-point scale, where 1 meant “strongly disagree” and 7 meant “strongly agree.” The measure of perceived effects of digital misinformation on others consisted of four parallel items with the same statement except replacing “I” and “my” with “the general others” and “their”. The three “self” items were averaged to create a measure of “perceived effects on oneself” (*M* = 3.98, *SD* = 1.49, *α* = 0.87). The three “others” items were also added and averaged to form an index of “perceived effects on others” (*M* = 4.62, *SD* = 1.32, *α* = 0.87).

#### The perceived self-other disparity (TPP)

TPP was derived by subtracting perceived effects on oneself from perceived effects on others.

#### Professional identification with medical community

Professional identification was measured using a three item, 7-point Likert-type scale (1 = *strongly disagree*, 7 = *strongly agree*) adapted from previous studies [65, 66] by asking respondents to indicate to what extent they agreed with the following statements: (1) I would be proud to be a medical staff member in the future; (2) I am committed to my major; and (3) I will be in an occupation that matches my current major. The three items were thus averaged to create a composite measure of professional identification (*M* = 5.34, *SD* = 1.37, *α* = 0.88).

#### Digital misinformation self-efficacy

Modified from previous studies [3], self-efficacy was measured with three items. Respondents were asked to indicate on a 7-point Linkert scale from 1 (strongly disagree) to 7 (strongly agree) their agreement with the following: (1) I think I can identify misinformation relating to COVID-19 on social media by myself; (2) I know how to verify misinformation regarding COVID-19 by using digital tools such as Tencent Jiaozhen^1^ and Piyao.org.cn^2^; (3) I am confident in my ability to identify digital misinformation relating to COVID-19. A composite measure of self-efficacy was constructed by averaging the three items (*M* = 4.38, *SD* = 1.14, *α* = 0.77).

#### Collectivism

Collectivism was measured using four items adapted from previous research [67], in which respondents were asked to indicate their agreement with the following statements on a 7-point scale, from 1 (strongly disagree) to 7 (strongly agree): (1) Individuals should sacrifice self-interest for the group; (2) Group welfare is more important than individual rewards; (3) Group success is more important than individual success; and (4) Group loyalty should be encouraged even if individual goals suffer. Therefore, the average of the four items was used to create a composite index of collectivism (*M* = 4.47, *SD* = 1.30, *α* = 0.89).

#### Intention to correct digital misinformation

We used three items adapted from past research [68] to measure respondents’ intention to correct misinformation on social media. All items were scored on a 7-point scale from 1 (very unlikely) to 7 (very likely): (1) I will post a comment saying that the information is wrong; (2) I will message the person who posts the misinformation to tell him/her the post is wrong; (3) I will track the progress of social media platforms in dealing with the wrong post (i.e., whether it’s deleted or corrected). A composite measure of “intention to correct digital misinformation” was constructed by adding the three items and dividing by three (*M* = 3.39, *SD* = 1.43,* α* = 0.81).

#### Intention to promote corrective information

On a 7-point scale ranging from 1 (very unlikely) to 7 (very likely), respondents were asked to indicate their intentions to (1) Retweet the corrective information about coronavirus on my social media account; (2) Share the corrective information about coronavirus with others through Social Networking Services. The two items were averaged to create a composite measure of “intention to promote corrective information” (*M* = 4.60, *SD* = 1.68, *r* = 0.77).

#### Control variables

We included gender, age, class (1 = undergraduate degree; 2 = master degree; 3 = doctoral degree), and clinical internship (0 = none; 1 = less than 0.5 year; 2 = 0.5 to 1.5 years; 3 = 1.5 to 3 years; 4 = more than 3 years) as control variables in the analyses. Additionally, coronavirus-related information exposure (i.e., how frequently they were exposed to information about COVID-19 on Weibo, WeChat, and QQ) and misinformation exposure on social media (i.e., how frequently they were exposed to misinformation about COVID-19 on Weibo, WeChat, and QQ) were also assessed as control variables because previous studies [69, 70] had found them relevant to misinformation-related behaviors. Descriptive statistics and bivariate correlations between main variables were shown in Table 1.

**Table 1:** Descriptive statistics and bivariate correlations

	**α**	**CR**	**AVE**	**PEDMO**	**PEDMOT**	**TPP**	**PIMC**	**DMSE**	**Collectivism**	**ICDM**	**IPCI**
**PEDMO**	.87	0.88	0.70	**0.84**							
**PEDMOT**	.87	0.88	0.72	.56^***^	**0.85**						
**TPP**	—	—	—	-.56^***^	.37^***^	—					
**PIMC**	.88	0.91	0.78	.14^***^	.20^***^	.04	**0.88**				
**DMSE**	.77	0.78	0.54	-.04	.08^**^	.12^***^	.25^***^	**0.73**			
**Collectivism**	.89	0.90	0.69	.04	.01	-.04	.28^***^	.23^***^	**0.83**		
**ICDM**	.81	0.81	0.59	.06^*^	-.02	-.08^**^	.07^**^	.20^***^	.18^***^	**0.77**	
**IPCI**	—	0.87	0.77	.10^***^	.08^**^	-.03	.22^***^	.30^***^	.26^***^	.47^***^	**0.88**

The square roots of AVE are shown in boldface on the diagonal

*PEDMO* Perceived Effects of Digital Misinformation on Oneself, *PEDMOT* Perceived Effects of Digital Misinformation on Others, *PIMC* Professional Identification with Medical Community, *DMSE* Digital Misinformation Self-efficacy, *ICDM* Intention to Correct Digital Misinformation, *IPCI* Intention to Promote Corrective Information

^*^p < .05, ^**^p < .01, ^***^p < .001

### Statistical analysis

We ran confirmatory factor analysis (CFA) in Mplus (version 7.4, Muthén & Muthén, 1998) to ensure the construct validity of the scales. To examine the associations between variables and tested our hypotheses, we performed structural equation modeling (SEM). Mplus was chosen over other SEM statistical package mainly because the current data set included some missing data, and the Mplus has its strength in handling missing data using full-information maximum likelihood imputation, which enabled us to include all available data [71, 72]. Meanwhile, Mplus also shows great flexibility in modelling when simultaneously handling continuous, categorical, observed, and latent variables in a variety of models. Further, Mplus provides a variety of useful information in a concise manner [73].

## Results

Table 2 shows the model fit information for the measurement and structural models. Five latent variables were specified in the measurement model. To test the measurement model, we examined the values of Cronbach’s alpha, composite reliability (CR), and average variance extracted (AVE) (Table 1). Cronbach’s alpha values ranged from 0.77 to 0.89. The CRs, which ranged from 0.78 to 0.91, exceeded the level of 0.70 recommended by Fornell (1982) and thus confirmed the internal consistency. The AVE estimates, which ranged from 0.54 to 0.78, exceeded the 0.50 lower limit recommended by Fornell and Larcker (1981), and thus supported convergent validity. All the square roots of AVE were greater than the off-diagonal correlations in the corresponding rows and columns [74]. Therefore, discriminant validity was assured. In a word, our measurement model showed sufficient convergence and discriminant validity.

**Table 2:** Summary of model fit for the hypothesized model

Model	χ^2^	*df*	χ^2^ /*df*	CFI	TLI	RMSEA	SRMR
Measurement model	291.80^***^	80	3.65	.98	.97	.04	.04
Structural model	305.97^***^	92	3.33	.98	.97	.04	.03

*CFI* comparative fit index, *TLI* Tucker–Lewis index, *RMSEA* root mean square error of approximation, *SRMR* standardized root mean squared residual

^***^p < .001

Five model fit indices–the relative chi-square ratio (χ^2^/*df*), the comparative fit index (CFI), the Tucker–Lewis index (TLI), the root mean square error of approximation (RMSEA), and the standardized root-mean-square residual (SRMR) were used to assess the model. Specifically, the normed chi-square between 1 and 5 is acceptable [75]. TLI and CFI over 0.95 are considered acceptable, SRMR value less than 0.08 and RMSEA value less than 0.06 indicate good fit [76]. Based on these criteria, the model was found to have an acceptable fit to the data.

**Fig. 2:**
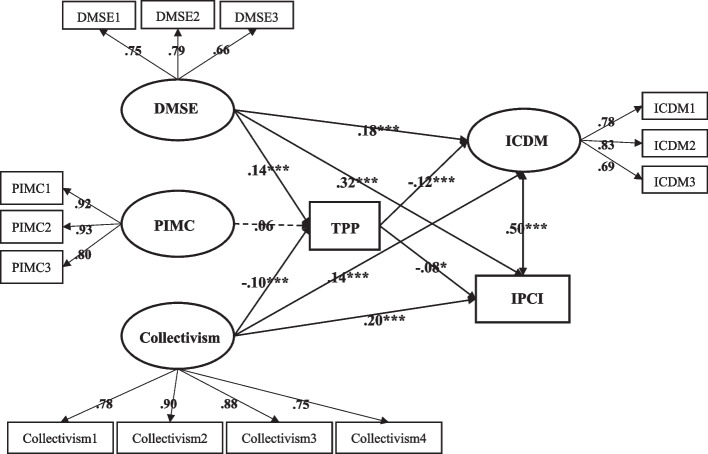
Note. *N* = 1,483. The coefficients of relationships between latent variables are standardized beta coefficients. Significant paths are indicated by solid line; non-significant paths are indicated by dotted lines. *p < .05, **p < .01; ***p < .001. DMSE = Digital Misinformation Self-efficacy; PIMC = Professional Identification with Medical Community; ICDM = Intention to Correct Digital Misinformation; IPCI = Intention to Promote Corrective Information

Figure 2 presents the results of our hypothesized model. H1 was rejected as professional identification failed to predict TPP (*β* = 0.06, *p* > 0.05). Self-efficacy was positively associated with TPP (*β* = 0.14, *p* < 0.001) while collectivism was negatively related to TPP (*β* = -0.10, *p* < 0.01), lending support to H2 and H3.

H4 posited that medical students with higher degrees of TPP would report greater intentions to correct digital misinformation. However, we found a negative association between TPP and intentions to correct misinformation (*β* = -0.12, *p* < 0.001). H4 was thus rejected. Regarding RQ1, results revealed that TPP was negatively associated with intentions to promote corrective information (*β* = -0.08, *p* < 0.05).

Further, our results supported H5 as we found that self-efficacy had a significant positive relationship with corrective intentions (*β* = 0.18, *p* < 0.001) and promotional intentions (*β* = 0.32, *p* < 0.001). Collectivism was also positively associated with intentions to correct misinformation (*β* = 0.14, *p* < 0.001) and promote corrective information (*β* = 0.20, *p* < 0.001), which answered RQ2.

Regarding RQ3 (see Table 3), TPP significantly mediated the relationship between self-efficacy and intentions to correct misinformation (*β* = -0.016), as well as the relationship between self-efficacy and intentions to promote corrective information (*β* = -0.011). However, TPP failed to mediate either the association between collectivism and corrective intentions (*β* = 0.011, *ns*) or the association between collectivism and promotional intentions (*β* = 0.007, *ns*).

**Table 3:** Indirect effects of self-efficacy and collectivism on intentions against misinformation through TPP

Path	Indirect path coefficient	95%CI	
		**LL**	**UL**
Self-efficacy → TPP → Correction	-.016	-.049	-.039
Self-efficacy → TPP → Promotion	-.011	-.047	-.037
Collectivism → TPP → Correction	.011	-.004	.002
Collectivism → TPP → Promotion	.007	-.006	.000

*CI* confidence interval, *LL* lower limit, *UL* upper limit

## Discussion

Recent research has highlighted the role of health professionals and scientists in the fight against misinformation as they are considered knowledgeable, ethical, and reliable [5, 77]. This study moved a step further by exploring the great potential of pre-professional medical students to tackle digital misinformation. Drawing on TPE theory, we investigated how medical students perceived the impact of digital misinformation, the influence of professional identification, self-efficacy and collectivism on these perceptions, and how these perceptions would in turn affect their actions against digital misinformation.

In line with prior studies [3, 63], this research revealed that self-efficacy and collectivism played a significant role in influencing the magnitude of third-person perception, while professional identification had no significant impact on TPP. As shown in Table 1, professional identification was positively associated with perceived effects of misinformation on oneself (*r* = 0.14, *p* < 0.001) and on others (*r* = 0.20, *p* < 0.001) simultaneously, which might result in a diminished TPP. What explains a shared or joint influence of professional identification on self and others? A potential explanation is that even medical staff had poor knowledge about the novel coronavirus during the initial outbreak [78]. Accordingly, identification with the medical community was insufficient to create an optimistic bias concerning identifying misinformation about COVID-19.

Our findings indicated that TPP was negatively associated with medical students’ intentions to correct misinformation and promote corrective information, which contradicted our hypotheses but was consistent with some previous TPP research conducted in the context of perceived risk [10, 79–81]. For instance, Stavrositu and Kim (2014) found that increased TPP regarding cancer risk was negatively associated with behavioral intentions to engage in further cancer information search/exchange, as well as to adopt preventive lifestyle changes. Similarly, Wei et al. (2008) found concerning avian flu news that TPP negatively predicted the likelihood of engaging in actions such as seeking relevant information and getting vaccinated. In contrast, the perceived effects of avian flu news on oneself emerged as a positive predictor of intentions to take protective behavior.

Our study shows a similar pattern as perceived effects of misinformation on oneself were positively associated with intentions to correct misinformation (*r* = 0.06, *p* < 0.05) and promote corrective information (*r* = 0.10, *p* < 0.001, See Table 1). While the reasons for the behavioral patterns are rather elusive, such findings are indicative of human nature. When people perceive misinformation-related risk to be highly personally relevant, they do not take chances. However, when they perceive others to be more vulnerable than themselves, a set of sociopsychological dynamics such as self-defense mechanism, positive illusion, optimistic bias, and social comparison provide a restraint on people’s intention to engage in corrective and promotional actions against misinformation [81].

In addition to the indirect effects via TPP, our study also revealed that self-efficacy and collectivism serve as direct and powerful drivers of corrective and promotive actions. Consistent with previous literature [61, 68], individuals will be more willing to engage in social corrections of misinformation if they possess enough knowledge, skills, abilities, and resources to identify misinformation, as correcting misinformation is difficult and their effort would not necessarily yield positive outcomes. Collectivists are also more likely to engage in misinformation correction as they are concerned for the public good and social benefits, aiming to protect vulnerable people from being misguided by misinformation [82].

This study offers some theoretical advancements. First, our study extends the TPE theory by moving beyond the examination of restrictive actions and toward the exploration of corrective and promotional actions in the context of misinformation. This exploratory investigation suggests that self-other asymmetry biased perception concerning misinformation did influence individuals’ actions against misinformation, but in an unexpected direction. The results also suggest that using TPP alone to predict behavioral outcomes was deficient as it only “focuses on differences between ‘self’ and ‘other’ while ignoring situations in which the ‘self’ and ‘other’ are jointly influenced” [83]. Future research, therefore, could provide a more sophisticated understanding of third-person effects on behavior by comparing the difference of perceived effects on oneself, perceived effects on others, and the third-person perception in the pattern and strength of the effects on behavioral outcomes.

Moreover, institutionalized corrective solutions such as government and platform regulation are non-exhaustive [84, 85]; it thus becomes critical to tap the great potential of the crowd to engage in the fight against misinformation [8] while so far, research on the motivations underlying users’ active countering of misinformation has been scarce. The current paper helps bridge this gap by exploring the role of self-efficacy and collectivism in predicting medical students’ intentions to correct misinformation and promote corrective information. We found a parallel impact of the self-ability-related factor and the collective-responsibility-related factor on intentions to correct misinformation and promote corrective information. That is, in a collectivist society like China, cultivating a sense of collective responsibility and obligation in tackling misinformation (i.e., a persuasive story told with an emphasis on collective interests of social corrections of misinformation), in parallel with systematic medical education and digital literacy training (particularly, handling various fact-checking tools, acquiring Internet skills for information seeking and verification) would be effective methods to encourage medical students to engage in active countering behaviors against misinformation. Moreover, such an effective means of encouraging social corrections of misinformation might also be applied to the general public.

In practical terms, this study lends new perspectives to the current efforts in dealing with digital misinformation by involving pre-professionals (in this case, medical students) into the fight against misinformation. As digital natives, medical students usually spend more time online, have developed sophisticated digital competencies and are equipped with basic medical knowledge, thus possessing great potential in tackling digital misinformation. This study further sheds light on how to motivate medical students to become active in thwarting digital misinformation, which can help guide strategies to enlist pre-professionals to reduce the spread and threat of misinformation. For example, collectivism education in parallel with digital literacy training would help increase medical students’ sense of responsibility for and confidence in tackling misinformation, thus encouraging them to engage in active countering behaviors.

This study also has its limitations. First, the cross-sectional survey study did not allow us to justify causal claims. Granted, the proposed direction of causality in this study is in line with extant theorizing, but there is still a possibility of reverse causal relationships. To establish causality, experimental research or longitudinal studies would be more appropriate. Our second limitation lies in the generalizability of our findings. With the focus set on medical students in Chinese society, one should be cautious in generalizing the findings to other populations and cultures. For example, the effects of collectivism on actions against misinformation might differ in Eastern and Western cultures. Further studies would benefit from replication in diverse contexts and with diverse populations to increase the overall generalizability of our findings.

## Conclusion

Drawing on TPE theory, our study revealed that TPP failed to motivate medical students to correct misinformation and promote corrective information. However, self-efficacy and collectivism were found to serve as direct and powerful drivers of corrective and promotive actions. Accordingly, in a collectivist society such as China’s, cultivating a sense of collective responsibility in tackling misinformation, in parallel with efficient personal efficacy interventions, would be effective methods to encourage medical students, even the general public, to actively engage in countering behaviors against misinformation.

## Data Availability

The datasets used and/or analyzed during the current study available from the corresponding author on reasonable request.

## Footnotes

1 Tencent Jiaozhen Fact-Checking Platform which comprises the Tencent information verification tool allow users to check information authenticity through keyword searching. The tool is updated on a daily basis and adopts a human-machine collaboration approach to discovering, verifying, and refuting rumors and false information. For refuting rumors, Tencent Jiaozhen publishes verified content on the homepage of Tencent's rumor-refuting platform, and uses algorithms to accurately push this content to users exposed to the relevant rumors through the WeChat dispelling assistant.

2 Piyao.org.cn is hosted by the Internet Illegal Information Reporting Center under the Office of the Central Cyberspace Affairs Commission and operated by Xinhuanet.com. The platform is a website that collects statements from Twitter-like services, news portals and China's biggest search engine, Baidu, to refute online rumors and expose the scams of phishing websites. It has integrated over 40 local rumor-refuting platforms and uses artificial intelligence to identify rumors.
